# Supportive breeding boosts natural population abundance with minimal negative impacts on fitness of a wild population of Chinook salmon

**DOI:** 10.1111/mec.12046

**Published:** 2012-10-01

**Authors:** Maureen A Hess, Craig D Rabe, Jason L Vogel, Jeff J Stephenson, Doug D Nelson, Shawn R Narum

**Affiliations:** * Columbia River Inter-Tribal Fish Commission, Hagerman Fish Culture Experiment Station3059F National Fish Hatchery Road, Hagerman, ID 83332, USA; † Department of Fisheries Resources Management, Nez Perce TribePO Box 1942, McCall, ID 83638, USA; ‡ Department of Fisheries Resources Management, Nez Perce TribePO Box 365, Lapwai, ID 83540, USA

**Keywords:** parentage analysis, reproductive success, salmonids, supplementation

## Abstract

While supportive breeding programmes strive to minimize negative genetic impacts to populations, case studies have found evidence for reduced fitness of artificially produced individuals when they reproduce in the wild. Pedigrees of two complete generations were tracked with molecular markers to investigate differences in reproductive success (RS) of wild and hatchery-reared Chinook salmon spawning in the natural environment to address questions regarding the demographic and genetic impacts of supplementation to a natural population. Results show a demographic boost to the population from supplementation. On average, fish taken into the hatchery produced 4.7 times more adult offspring, and 1.3 times more adult grand-offspring than naturally reproducing fish. Of the wild and hatchery fish that successfully reproduced, we found no significant differences in RS between any comparisons, but hatchery-reared males typically had lower RS values than wild males. Mean relative reproductive success (RRS) for hatchery F_1_ females and males was 1.11 (*P* = 0.84) and 0.89 (*P* = 0.56), respectively. RRS of hatchery-reared fish (*H*) that mated in the wild with either hatchery or wild-origin (*W*) fish was generally equivalent to *W* × *W* matings. Mean RRS of *H* × *W* and *H* × *H* matings was 1.07 (*P* = 0.92) and 0.94 (*P* = 0.95), respectively. We conclude that fish chosen for hatchery rearing did not have a detectable negative impact on the fitness of wild fish by mating with them for a single generation. Results suggest that supplementation following similar management practices (e.g. 100% local, wild-origin brood stock) can successfully boost population size with minimal impacts on the fitness of salmon in the wild.

## Introduction

Artificial breeding programmes are widely used for the conservation of threatened or endangered species and for the restoration of declining populations (IUCN 1998; Frankham *et al*. 2002; Fraser 2008). Conditions associated with artificial rearing, such as the absence of predators, food availability and disease treatments, result in selective pressures that are widely different from natural environments. Artificially reared organisms are thus subject to adaptation to captivity (i.e. domestication selection; Frankham *et al*. 2002; Ford *et al*. 2008). Large-scale, human-mediated releases of plants and animals occur worldwide, and when artificially reared individuals are released to the wild, there can be negative genetic effects on native or wild populations (reviewed in Laikre *et al*. 2010). Specifically, considerable concern exists over domestication selection because reproductive fitness of wild populations can be reduced when artificially reared individuals mate with wild counterparts (Araki *et al*. 2009). Additionally, gene flow from these individuals into native or wild populations can homogenize genetic structure of wild populations (Eldridge *et al*. 2009) and disrupt the capacity of natural populations to adapt to changing environmental conditions (McGinnity *et al*. 2009).

Hatchery-reared Pacific salmon and steelhead (*Oncorhynchus spp*.) are commonly released into the wild environment to boost abundance of declining populations, mitigate for environmental and habitat disturbances and to enhance harvest fisheries. Salmonid hatcheries are broadly classified by having conservation or harvest objectives (reviewed in Naish *et al*. 2007). Traditional salmonid hatchery programmes with harvest objectives are designed to increase the population census size using hatchery-origin fish that are reared for multiple generations in an artificial environment, and often with out-of-basin (i.e. nonlocal) brood stock that may not be locally adapted to environmental conditions. Due to the nature of traditional hatchery programmes, fish are subject to negative genetic impacts such as inbreeding (reviewed in Wang *et al*. 2002), domestication selection (Heath *et al*. 2003; Reisenbichler *et al*. 2004; Christie *et al*. 2011) and reduced fitness due to repeated generations in captivity (Araki *et al*. 2007a). In contrast, supplementation programmes are designed to mitigate for ongoing limiting factors to survival (i.e. dams, removal of individuals in harvest fisheries, habitat degradation, etc.) with the goal of increasing natural population size for conservation and population recovery purposes, while striving to minimize the genetic impact to natural populations (Cuenco *et al*. 1993; Waples *et al*. 2007). Integrating wild-origin individuals into supplementation brood stock is one method that can be used to help offset potential negative effects on fitness (Wang & Ryman 2001; Duchesne & Bernatchez 2002; Ford 2002). Artificially produced offspring from brood stock (either hatchery or wild-origin) are subsequently released into the wild to spawn. This approach has caused some concern because the artificial environment can select for individuals that may be poorly adapted to the natural environment (Johnsson *et al*. 1996; Pearsons *et al*. 2007; Frankham 2008; Christie *et al*. 2011), and hatchery-reared fish may impose negative impacts to the fitness of wild fish (Araki *et al*. 2009).

The concern over hatchery fish spawning in the wild is supported by theoretical work that shows that even if local, wild-born fish are used for brood stock each year, domestication selection in the hatchery could lead to fitness consequences for the wild population (Lynch & O'Hely 2001; Ford 2002; Goodman 2005; Chilcote *et al*. 2011). However, additional studies demonstrate that increasing the proportion of wild-born individuals into the captive population can slow the rate of genetic adaptation to captivity (Frankham & Loebel 1992) and reduce inbreeding in supplementation programmes (Duchesne & Bernatchez 2002). Empirical studies have shown that hatchery-reared salmonids have lower reproductive success in the wild compared with wild-origin fish in the first generation (Araki *et al*. 2007b; Williamson *et al*. 2010; Berntson *et al*. 2011; Theriault *et al*. 2011; Anderson *et al*. 2012), but few studies have investigated fitness effects over multiple generations. Two recent studies that examined fitness over two generations focused on a single population of steelhead trout (*Oncorhynchus mykiss*) and demonstrated that an increased number of generations in captivity can have negative fitness consequences on the population, but results were highly variable across years (Araki *et al*. 2007a, 2009). Fitness declines of hatchery-reared fish in the wild have been attributed to a number of causes. Hypotheses include the absence of sexual selection in the hatchery environment (stronger effect on hatchery males than females—Theriault *et al*. 2011; Anderson *et al*. 2012), the use of nonlocal origin brood stock over multiple generations (Chilcote *et al*. 1986; McLean *et al*. 2003; Araki *et al*. 2007b), differences in spawning location and age (Williamson *et al*. 2010), as well as body size, return date and the number of same-sex competitors (Berntson *et al*. 2011). Despite evidence that hatchery-reared fish can have lower reproductive success in the wild compared with their wild-origin counterparts, the potential for benefits from supplementation programmes using local-origin fish for brood stock warrants more extensive study. Specifically, when hatchery-reared fish are allowed to spawn naturally, can supportive breeding boost abundance while minimizing negative fitness impacts on wild fish?

Despite the need for this type of evaluation of supplementation programmes, all published studies evaluating reproductive success of hatchery-reared salmonids in the natural environment focus on programmes that use both wild and hatchery-reared fish as brood stock, and supplementation was initiated prior to the study of the target programme. In addition, studies have largely been focused on steelhead, which are typically reared in the hatchery to smolt within 1 year before being released as juveniles, rather than rearing to age 2 or older as typically found in nature (Araki *et al*. 2007a,b, 2009; Berntson *et al*. 2011). Recent studies are available for a few other salmonids (Berejikian *et al*. 2009, chum salmon; Williamson *et al*. 2010 and Anderson *et al*. 2012, Chinook salmon; Theriault *et al*. 2011, coho salmon), but none have estimated lifetime relative reproductive success (RRS) over multiple generations in the wild. Thus, there is a need for greater species coverage as well as multi-generation studies that examine supportive breeding programmes from the initiation of supplementation. Further, additional studies of Chinook salmon (*Oncorhynchus tshawytscha*) in natural environments may be critical because of the extensive use of hatchery supplementation for this species and the potential for relatively high fitness of hatchery-reared fish of this species (Schroder *et al*. 2008, 2010). The available RRS studies on Chinook salmon in the wild evaluate adult to juvenile production (Williamson *et al*. 2010) and colonization of newly accessible habitat (Anderson *et al*. 2012), and no published RRS studies have evaluated the lifetime fitness (adult to adult) of this species over multiple generations.

Here, we assess the lifetime fitness of Chinook salmon in Johnson Creek, a tributary to the South Fork Salmon River (SFSR) in central Idaho, USA, by following an ongoing supplementation programme for two generations (1998–2010), beginning with the first year (1998) that wild-origin returns were taken into the hatchery and used for brood stock. We use genetic parentage assignments to test the following: (i) Does the hatchery programme provide a demographic boost to the wild population over two generations? (ii) Are there differences in reproductive success between wild and hatchery-reared fish spawning in nature? (iii) Are there short-term (approximately two generations) genetic consequences of supplementation—that is, do hatchery-reared fish spawning in nature reduce the fitness of the wild population?

## Methods

### Study site and sample collection

**Fig 1:**
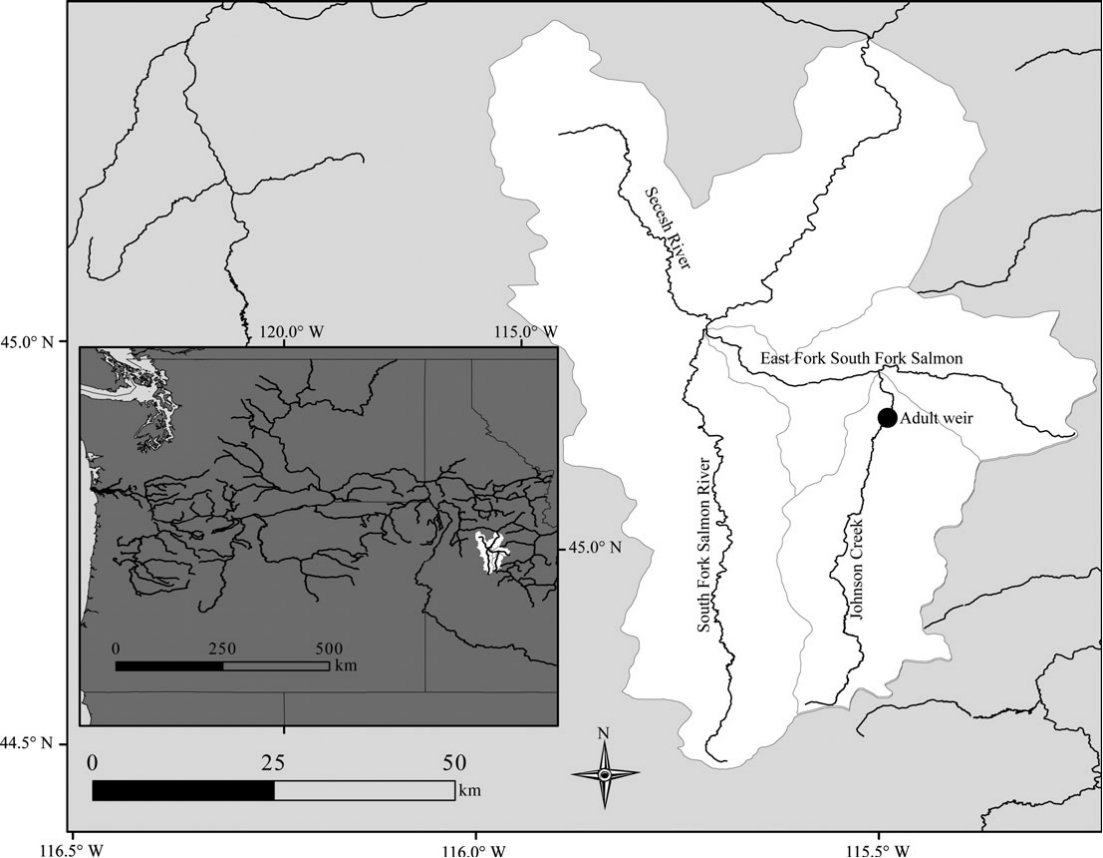
Map of the study area, showing location of the weir. Inset map shows the location of the South Fork Salmon River basin highlighted in white.

The Salmon River basin is one of the largest subbasins of the Columbia River and covers approximately 36 000 thousand square kilometres within the Northern Rocky Mountains of central Idaho. The Interior Columbia Technical Recovery Team (ICTRT) identified three unique populations of spring/summer Chinook salmon that occur within the SFSR: the SFSR mainstem, the Secesh and the East Fork SFSR. Johnson Creek is the primary spawning aggregate of Chinook salmon within the East Fork SFSR (Fig. 1) and represents one of 32 spring/summer Chinook salmon populations listed under the Endangered Species Act in the Snake River Evolutionarily Significant Unit (ICTRT 2005). The putative wild Chinook salmon population aggregations in these three areas of the SFSR remain intact despite substantial releases of hatchery stock for supplementation and harvest augmentation in the SFSR mainstem (Matala *et al*. 2012). A supplementation programme was initiated in 1998 by the Nez Perce Tribe in an effort to prevent extirpation by increasing natural production of Chinook salmon in Johnson Creek.

Tissue samples and associated biological data were collected from 7726 returning adults encountered at the Johnson Creek picket-style weir, and during annual multiple-pass spawning ground surveys conducted upstream and downstream of the weir from 1998 to 2010. The weir occurs downstream of approximately 94% of the spawning habitat (Rabe & Nelson 2010). In the field, gender was determined by physical morphology, fork length was measured to the nearest centimetre, and origin was identified through the presence/absence of marks, tags or clips (hatchery fish have a coded wire tag and/or a visual implant elastomer tag; hatchery strays from other locations have adipose fins removed). If a fish had no visible mark, it was inferred to be produced in the wild. A tissue sample from the caudal fin was taken for genetic analysis, and these individuals were marked with an individually numbered operculum disk tag. Nontagged fish were sampled on multiple-pass spawning ground surveys upstream and downstream of the weir to achieve a high sampling rate over the course of the study (78–100%; annual mean = 95%). Only wild-origin (*W*, defined as fish born and reared in the natural environment, regardless of parentage), returning adults were selected for brood stock each year; all wild adults not collected for brood stock and all hatchery-origin adults were released upstream of the weir to spawn naturally. The actual genetic composition of fish used for brood stock was 98% wild origin because a total of seven hatchery-reared fish over the period of 2001 through 2005 were unintentionally used as brood stock (5 fish from brood year, BY, 1998 and 2 fish from BY 2000). Hatchery smolts were released directly into Johnson Creek after rearing in a hatchery environment for 18 months. No fish were collected as brood stock in 1999 because only 22 fish returned, and all were allowed to spawn naturally.

**Fig 2:**
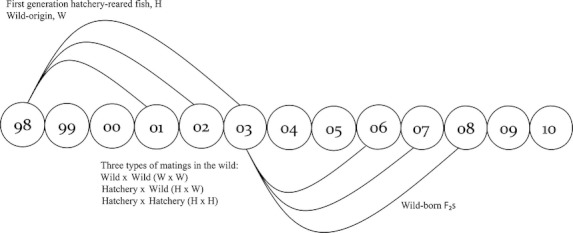
Sampling design for the study. Illustrated is the sampling design for the first year of supplementation in 1998, but the same design applies to annual brood stock collections for 2000 to 2005 (5-year-olds from brood year, BY 2005 return in 2010, the last sampling year of this study). Circles represent the BY, corresponding to the year that adults return to Johnson Creek to spawn. This example shows first-generation hatchery fish (F_1_) from BY 1998, which return to spawn alongside their wild-origin counterparts in 2001 (age 3, ‘jacks’), 2002 (age 4) and 2003 (age 5). Mating among hatchery-reared and wild-origin fish occurred in every year beginning in 2001 to create wild-born F_2_s, which return 3–5 years later. The example follows age 5 fish (born in 1998) that returned as adults in year 2003 and produced wild-born fish (F_2_s) that returned in years 2006 through 2008.

The proportion of returns by age class to Johnson Creek varied between hatchery-reared and wild-origin fish. The majority of wild-origin fish returned at age 4 (mean, 62%), followed by age 5 (mean, 28%), and a smaller proportion returned at age 3 that were exclusively males (termed ‘jacks’; mean, 10%). Most hatchery-reared fish returned to Johnson Creek at age 3 (mean, 43%, all males) and 4 (mean, 49%); with a smaller proportion that returned at age 5 (mean, 8%). Adult offspring from the first year of supplementation (BY 1998) returned to Johnson Creek at ages 3, 4 and 5 in 2001, 2002 and 2003, respectively. All returning F_1_ hatchery-reared fish (*H*) were released upstream of the weir for natural spawning with their wild F_1_ counterparts (Fig. 2). Offspring that resulted from naturally spawning F_1_s from BY 1998 (first year of supplementation) were termed F_2_ and returned to the Johnson Creek weir as adults in 2004 to 2008 (Fig. 2). The same type of sampling scheme was achieved in each return year through 2005, as the last of the offspring (5-year-olds) from BY 2005 returned in 2010. Genetic parentage analysis was used to assign wild-origin F_2_ returns back to their F_1_ parents.

### Parentage analysis

Genomic DNA was extracted from fin tissue following manufacturer's protocols for QIAGEN DNeasy extraction kits, and individuals were genotyped using 15 microsatellite loci: *Ots100* (Nelson & Beacham 1999), *Ots3M* (Greig & Banks 1999), *Ssa408* (Cairney *et al*. 2000), *OMM*1080 (Rexroad *et al*. 2001), *Ots*211, *Ots*212, *Ots*213, *Ots*201b, *Ots*208b (Greig *et al*. 2003), *Ots*G474, *Ots311* (Williamson *et al*. 2002), *Ogo*2, *Ogo*4 (Olsen *et al*. 1998), *Ots*9 (Banks *et al*. 1999) and *Oki100* (K. Miller, unpublished data). Markers were amplified and genotyped as described by Narum *et al*. (2010). Briefly, fluorescently labelled PCR products were separated with fragment analysis chemistry on an Applied Biosystems 3730 Genetic Analyzer and genotyped with GeneMapper software. MSExcel Microsatellite toolkit was used to identify duplicate genotypes. Duplicates resulted from fish sampled first at the weir, and again on a redd or spawning ground survey. Use of operculum tags to mark fish at the weir minimized the occurrence of duplication to 58 individuals, and in each of these cases, only the first capture sample at the weir was included in the analysis.

To assign returning adult offspring to parent(s), we used an exclusion approach with the program cervus 3.0 (Marshall *et al*. 1998; Kalinowski *et al*. 2007). Individuals genotyped for at least 12 of the 15 loci were included in parentage analyses. For single-parent-offspring comparisons, only those exhibiting no mismatches at a minimum of 14 common loci were considered true parent-offspring groupings. Only one mismatching locus was allowed for trios (offspring matching two parents), with at least 12 loci in common among all three individuals. These thresholds were highly conservative to avoid false assignments, and genotyping error was estimated to be very low at <1% based on concordance of quality control tests with repeated genotyping using approximately 5% of the samples; however, this approach may not account for all potential errors in the study. Returning F_1_ offspring (*W* and *H*) were assigned to parents for each BY from 1998 to 2005 (with the exception of BY 1999 hatchery-reared parents, described above). For example, F_1_ offspring (*W* and *H*) from BY 1998 returned in years 2001 through 2003 (Fig. 2). Specifically, salmon returning in 2001 through 2003 were tested against biologically plausible candidate parents (i.e. BY 1998). Following our second and third objectives, respectively, F_2_ offspring were assigned to F_1_ parents in two ways: (i) Second-generation (F_2_) offspring returning in years 2004–2010 were assigned to F_1_ parents from BY 1998 and 2000 (i.e. F_2_ are the grand-offspring of F_0_ fish that spawned in 1998 and 2000). This allowed us to specifically follow two initial brood years of supplementation through the second generation. (ii) Second-generation (F_2_) offspring returning in 2006–2010 were assigned to F_1_ parents that spawned naturally in 2003–2005. This also allowed us to follow the second-generation returns, however, targeting combined age groups in each of these F_1_ brood years increased our sample size and allowed direct comparison to published literature (Araki *et al*. 2009) and allowed for evaluation of genetic impacts to wild fish when hatchery fish mate with them. These brood years were chosen because all parents and offspring were sampled during the years of our study.

We empirically evaluated parentage assignment error rate by attempting to assign offspring returning in 2001 to 2005 to parents used for brood stock in 1998 and 2000. Parentage assignment errors fall into two categories: type A and B errors (different from Type I and II statistical errors; Araki & Blouin 2005). The failure to assign a true parent when that parent is in the sample, type A error, was determined by first attempting to assign hatchery-reared offspring to parents that were used for brood stock (all hatchery-reared fish should assign to a parent). Specifically, we evaluated offspring that assigned to parent pairs (or 2 of 2 brood stock parents) because we have no way of validating the single-parent assignments from hatchery mating records. We then calculated concordance between the parentage assignment results and the mated parents indicated by hatchery records; an error was recorded if a hatchery-reared fish did not assign to a parent or if it assigned to parents that did not match hatchery mating records. Type B error, assignment to an untrue parent (occurs when the true parent is absent or when the true parent is present but failed to be assigned), was calculated by attempting to assign wild-origin fish to parents that were used for brood stock (no wild-origin fish would have brood stock parents) and attempting to assign hatchery-reared fish to parents not used for brood stock. The stringency of the parentage assignment criteria used influences type A and type B errors as described in Araki & Blouin (2005). Specifically, Araki & Blouin (2005) found that type B error in their data set for steelhead was 1.4% when no mismatches were allowed, but jumped up to 30.5% when two mismatches were allowed. Because type B error is used to calculate unbiased RRS, minimizing this error ensures the minimum bias on RRS.

### Relative reproductive success

Using parentage analysis, we estimated lifetime reproductive success, that is, the number of returning adult offspring produced per adult individual. Lifetime reproductive success was estimated for F_0_ fish that produced F_1_s in the hatchery and in the wild and estimated for returning adult F_1_ fish that produced adult F_2_ offspring in the natural environment. Using our empirically derived type B error rate, we obtained unbiased estimates of RRS following equation 14 from Araki & Blouin (2005). RRS estimates were not corrected for effects of harvest because there is no differential harvest between hatchery and wild fish (Johnson Creek hatchery fish are not adipose marked; therefore, there is no influence of a mark selected fishery).

To address our first objective and determine whether the supplementation programme provided a demographic boost to the natural population, we compared the numbers of offspring produced by fish that were removed from the wild and taken into the hatchery intended for use as brood stock versus individuals that were allowed to spawn in the natural environment (BY 1998–2005, with exception of BY 1999; Table 1). The numbers of adult offspring produced each year (1998–2005) and the numbers of adult grand-offspring produced from BY 1998 and BY 2000 were calculated based on parentage exclusion results for both artificially and naturally spawning individuals. Not all fish taken for brood stock had the opportunity to contribute offspring to the next generation due to prespawn mortality, unsuccessful spawning or culling of eggs to prevent disease. In addition, not all individuals had complete genetic data; therefore, some parent–offspring relationships were not possible to detect in our analyses. To take the most conservative approach, we counted all potential parents that were removed at the weir for brood stock, even if they did not have the opportunity to contribute offspring. We also counted all potential parents that were sampled regardless of the completeness of genetic data.

**Table 1:** Comparison of the number of returning adult offspring (including jacks) produced by fish removed at the weir for hatchery brood stock and the number of returning adult offspring produced by fish allowed to spawn in the natural environment

Brood year	*n*, Brood stock	*n*, Natural spawners	Hatchery produced adult offspring relative to wild
1998	55	104	2.77
1999	0	22	n/a
2000	72	87	1.22
2001	147	1334	5.35
2002	96	1103	5.48
2003	79	715	8.01
2004	57	271	5.29
2005	75	123	4.70
Mean			4.69

*n* is the sample size for the number of wild fish removed at the weir intended for use as brood stock (even if they did not have the opportunity to contribute offspring to the next generation), and the number of wild and hatchery fish allowed to spawn in the natural environment. Both *n* categories represent all individuals that were sampled, regardless of the occurrence of incomplete genetic data.

Our second objective was to determine whether there were differences in reproductive success between hatchery-reared and wild-origin fish spawning naturally (reproductive success of F_1_ fish produced from BY 1998 and 2000). Mean reproductive success was estimated separately for males and females by age class. First-generation (F_1_) offspring from BYs 1998 and 2000 returned as jacks (age 3 males) in 2001 and 2003, and F_1_ males and females (ages 4 and 5) returned in 2002 through 2005 (Fig. 2). To compare reproductive success separately for jacks, males and females in each year, we calculated RRS by dividing the average reproductive success of hatchery-reared fish by the average reproductive success of wild fish of the same gender and age. RRS estimates were calculated in two ways to include (i) all F_1_ potential parents and (ii) only successful F_1_ parents that contributed to the next generation by producing one or more returning adult offspring. To compare reproductive success of hatchery-reared males and females, we calculated RRS by dividing the average reproductive success of hatchery-reared males by the average reproductive success of hatchery-reared females of the same age.

Finally, to assess the effect of hatchery-reared fish on the fitness of wild-origin fish, we compared the reproductive success among mating types in the wild for BY 2003 to 2005 (*H* × *H*, *H* × *W*, *H* × – vs. *W* × *W* and *W* × –; where ‘–’ equals one unknown/unassigned parent). Age classes were combined in each return year (i.e. RS of all returns in a given year was evaluated), but comparisons were made separately for males and females in addition to an analysis of sexes combined (Table 3). If hatchery rearing reduces the fitness of wild-origin fish, we would expect the *H* × *W* mating type to produce significantly fewer returning adult offspring than the *W* × *W* mating type.

We tested statistical significance of all RRS estimates with a two-tailed permutation procedure using the comparison of means algorithm applied in perm 1.0 (Duchesne *et al*. 2006) set at 10 000 permutations. To evaluate the power of our analysis, we used the distribution of reproductive success differences from the permutation tests to calculate the minimum difference in reproductive success that we could detect with 80% and 95% probability. Overall RRS values were estimated by weighted geometric means (by number of offspring), and corresponding *P*-values were calculated on the basis of Fisher's combined probability.

## Results

### Parentage analysis

Combined nonexclusion probability for assignment of the first parent, second parent and parent pair was 2.30E−07, 2.91E−10 and 2.25E−17, respectively (Table S1, Supporting information). Approximately 97.6% of samples (7481 of 7668; Table S2, Supporting information) were successfully genotyped at 12 or more loci and were included in parentage analysis. Of the adult offspring returning in 2001–2010 (representing BY 1998–2005), 87% on average were assigned a single parent or parental pair, with assignment success ranging from 69% in return year 2003 to 95% in 2005. Lower weir efficiencies (i.e. sampling rate of returning potential parents) in the initial years of the study (mean weir efficiency for 1998 and 2000 was 63%) likely influenced the assignment success rate. Improvements made to weir operation were accompanied by parentage assignment success rates consistently >90% beginning for fish returning in 2005 through 2010. Distribution of the number of offspring produced by fish that returned to spawn in the wild in 1998 through 2005 was highly skewed. The majority of natural spawners (both hatchery-reared and wild) produced no adult offspring, and approximately 32% of all females produced one or more returning adult offspring (Fig. S1, Supporting information). Only 16% of hatchery males produced adult offspring compared with 25% of wild males (mean for 1998 through 2005). The number of hatchery-reared and wild-origin F_1_ counterparts (born in 1998 and 2000) that returned and successfully reproduced in years 2001 through 2005 is shown in Table 2, and the number of F_2_ fish that hatched in the wild in BYs 2003 to 2005 is shown in Table.

**Table 2:** Relative reproductive success (RRS) of successful (produced at least one returning adult offspring) female, male and jack F_1_ fish from brood year (BY) 1998 and 2000

Return year	*n* F1 (*H*/*W*)	RS Hatchery	Variance hatchery	RS Wild	Variance wild	RRS*	*P*-value	80%/95% Power†	Age of returns
Females (4- & 5-year-old)									
2002	29/13	1.21	0.31	1.23	0.19	0.98	1.00	0.84/0.75	4 year from BY 1998
2003	20/43	1.25	0.20	1.30	0.41	0.96	0.83	0.85/0.76	5 year from BY 1998
2004	32/32	3.19	3.64	2.63	4.50	1.22	0.30	1.24/1.36	4 year from BY 2000
2005	8/3	4.25	1.07	5.00	9.00	0.85	0.55	0.85/0.58	5 year from BY 2000
Overall female‡						1.11	0.84		
Males (4- & 5-year-old)									
2002	24/32	1.21	0.26	1.25	0.39	0.97	0.83	0.85/0.74	4 year from BY 1998
2003	6/28	1.67	0.67	1.36	0.61	1.23	0.39	1.37/1.53	5 year from BY 1998
2004	26/36	2.54	4.34	3.17	4.43	0.80	0.27	0.78/0.66	4 year from BY 2000
2005	0/0	—	—	—	—	—	—	—	5 year from BY 2000
Overall male						0.89	0.56		
Jacks (3-year-old)									
2001	10/0	1.10	0.10	—	—	—	—	—	3 year from BY 1998
2003	15/8	1.20	0.31	1.75	1.07	0.68	0.16	0.88/0.66	3 year from BY 2000
Overall jack					—	—	—		

*n* is the sample size for number of naturally spawning successful (produced one or more returning adult offspring) hatchery-reared and wild F_1_ fish from BY 1998 and BY 2000.

* RRS is calculated as the RS of hatchery-reared fish over the RS of wild-origin fish, and associated *P*-values are based on two-tailed permutation tests. Overall RRS was estimated using weighted geometric means, and the according *P*-values were calculated.

† Statistical power is the RRS value that would be significant with 80% and 95% probability.

‡ Overall RRS estimate for females does not include return year 2005 due to low sample size.

**Table 3:** Relative reproductive success (RRS) of naturally spawning F_1_ parents by mating type

Return year	*n* F_2_ offspring assigned	RRS*	*P*-value	80%/95% Power†
*H* × *H* vs. *W* × *W*				
Females				
2003	4/62	0.87	0.83	0.87/0.43
2004	40/79	0.76	0.17	0.76/0.67
2005	30/22	1.14	0.67	1.36/1.55
Overall female		0.87	0.58	
Males				
2003	4/62	1.03	1.00	1.31/1.58
2004	40/79	0.94	0.76	0.77/0.67
2005	30/22	1.02	1.00	1.50/1.74
Overall male		0.98	1.00	
Overall both sexes		0.94	0.95	
*H* × *W* vs. *W* × *W*				
Females				
2003	41/62	1.05	0.68	1.13/1.18
2004	108/79	1.12	0.48	1.21/1.32
2005	68/22	1.30	0.33	1.35/1.49
Overall female		1.14	0.62	
Males				
2003	41/62	0.96	0.85	0.88/0.80
2004	108/79	1.08	0.67	1.21/1.31
2005	68/22	0.93	0.83	0.69/0.51
Overall male		1.00	0.96	
Overall both sexes		1.07	0.92	
*H* × – vs. *W* × –				
Females				
2003	4/10	0.90	1.00	0.78/0.78
2004	5/15	0.72	0.77	0.63/0.41
2005	6/7	0.85	1.00	0.86/0.57
Overall female		0.82	1.00	
Males				
2003	1/4	—	—	—
2004	5/9	1.31	0.65	1.44/1.67
2005	2/8	0.75	1.00	0.75/0.75
Overall male		1.06	0.93	
Overall both sexes		0.91	1.00	

*n* is the sample size for the number of wild-born F_2_ offspring that assigned to each parental mating type.

* RRS is calculated as the RS of hatchery-reared fish over the RS of wild-origin fish, and associated *P*-values are based on two-tailed permutation tests. Overall RRS was estimated using weighted geometric means, and the according *P*-values were calculated on the basis of Fisher's combined probability.

† Statistical power is the RRS value that would be significant with 80% and 95% probability.

No offspring were compatible with more than one set of parents. There were 36 (0.9% of parentage assignments) offspring that assigned to a single parent in 1 year (with zero mismatches) and assigned to a parental pair in a different year. In these few cases, the assignment to two parents was accepted given the lower value of the combined nonexclusion probability of parent pairs compared with single-parent assignments. Approximately 5% of the parentage assignments were not logically possible, the majority of which occurred in the first supplementation year, 1998. In the cases where ‘wild’ offspring assigned to parent pairs that were mated in the hatchery, these offspring (*n* = 97, 80% were from BY 1998) were treated as hatchery-reared in subsequent RRS analyses because their hatchery mark was likely not observed during field sampling. A total of 125 offspring were not counted in RRS estimates. Specifically, 56 ‘wild’ offspring assigned to a brood stock parent and a naturally spawning parent, 63 ‘wild’ offspring assigned to a single brood stock parent, and 6 ‘hatchery’ offspring assigned to parents that were not used for brood stock. A small opportunity exists for spawning downstream of the weir, and these particular types of matings (brood stock × natural spawner) may have occurred in low numbers before one parent was taken into the hatchery. For example, there were 20 ‘wild’ offspring from BY 1998 that assigned to two parents, where one parent was removed at the weir for brood stock, and the other parent was a natural spawner. These 20 offspring had one male parent in common that mated with multiple females (not used for brood stock). The male parent in this case successfully mated downstream of the weir before being captured for brood stock. These instances were not included in error estimates, and likewise these particular offspring were not included in RRS estimates.

For the empirical evaluation of parentage assignment errors, we found that all hatchery-reared offspring (identified via coded wire tags and/or visual implant elastomer tags) were assigned to parents that were used as brood stock, but 3.5% did not assign to the known mated parent pairs indicated by hatchery records (type A error). Inaccurate hatchery records cannot be distinguished from parentage errors and were therefore included in error estimates. Assignment of offspring to an untrue parent(s) resulted in overall 2.0% type B error (78 of 3933 offspring assigned to untrue parents). Specifically, 3.0% of hatchery-reared offspring assigned to one parent not used for brood stock, and 1.6% of wild-origin offspring assigned to one parent used for brood stock. Type B errors were confined to single-parent assignments only, as there were no trios.

### Relative reproductive success

#### Demographic boost from hatchery-reared fish?

The numbers of returning adult offspring produced by fish removed for brood stock compared with their naturally spawning counterparts were variable each year. A range of 1.22 (BY 2000) to 8.01 (BY 2003) times as many returning adult offspring were produced in the hatchery compared with in the wild (Table 1). Averaged across all seven brood years, fish removed for brood stock produced 4.69 times more returning adult offspring (average for BY 1998 and BY 2000: 2.00) and 1.32 times as many returning adult grand-offspring on average for two brood years (BY 1998: 1.37; and 2000: 1.28) compared with their naturally spawning counterparts. Even though survival advantages of the hatchery environment were no longer present in the second generation (as these fish produced offspring in the wild environment), the demographic boost provided by the hatchery from BY 1998 and BY 2000 continued in the second generation.

#### Differences in hatchery-reared versus wild-origin reproductive success?

Estimates of RRS for hatchery-reared and wild-origin naturally spawning F_1_ offspring (from BYs 1998 and 2000) are shown separately for jacks, males and females by age class in Table S3 (Supporting information, for all potential parents) and Table 2 (for successful spawners only). For hatchery-reared F_1_ females, mean RRS = 1.00 (*P* = 0.19), and none of the comparisons were significantly different from 1.0 (Table S3, Supporting information). For hatchery-reared adult males, mean RRS = 0.64 (*P* < 0.01) and was significantly lower in 2002 and for the 3 years combined (Table S3, Supporting information). Only one jack year was compared because wild-origin jacks that returned in 2001 did not produce any adult offspring. Unbiased RRS for hatchery-reared jacks in 2003 was 0.32 and was significantly lower (*P* < 0.01) than wild-origin counterparts (Table S3, Supporting information). The age 5 offspring from BY 2000 were not included in overall RRS estimates due to small sample size (0 males and only 12 females returned in 2005). Hatchery-reared male to hatchery-reared female RRS was 0.54 (*P* = 0.03, age 4 from BY 1998) in 2002, 1.21 (*P* = 0.77, age 5 from BY 1998) and 0.60 (*P* = 0.03, age 4 from BY 2000) in 2004.

**Fig 3:**
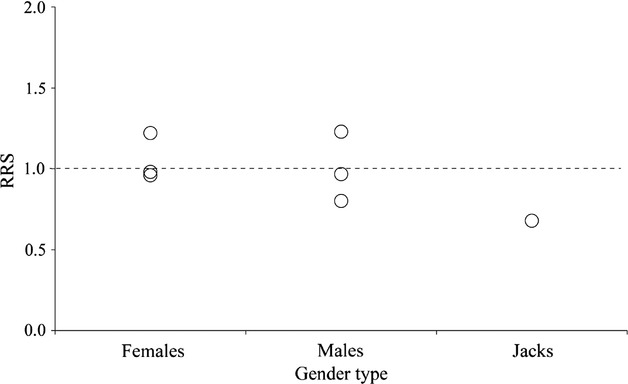
Relative reproductive success (RRS) of successful F_1_ spawners that produced one or more adult offspring (from BY 1998 and 2000), hatchery-reared relative to wild-origin fish for each gender type. Each point represents the estimate of RRS for each year compared and used to quantify overall RRS estimates; 2002–2004 (see associated Table 2). The dotted line (RRS = 1.0) represents where reproductive success of hatchery-reared fish is equal to that of wild-origin fish. Jacks are 3-year-old males.

In F_1_ return years 2002–2004 (from BY 1998 and BY 2000), 40% of wild males and 31% of hatchery-reared males produced at least one adult offspring; 45% of wild females and 41% of hatchery-reared females produced at least one adult offspring (Table S4, Supporting information). Of the wild and hatchery fish that successfully reproduced (i.e. one or more adult offspring), RRS estimates were very similar and not statistically significant between any comparisons (Table 2; Fig. 3). For hatchery-reared F_1_ females, unbiased RRS ranged from 0.96 (*P* = 0.83) to 1.22 (*P* = 0.30), and mean RRS = 1.11 (*P* = 0.84). For hatchery-reared F1 males, unbiased RRS ranged from 0.80 (*P* = 0.27) to 1.23 (*P* = 0.39), and mean RRS = 0.89 (*P* = 0.56). Unbiased RRS for hatchery-reared jacks in 2003 was 0.68, but was not significantly lower (*P* = 0.16) than wild-origin counterparts (Table 2; Fig. 3).

#### Hatchery impacts to fitness of wild fish?

**Fig 4:**
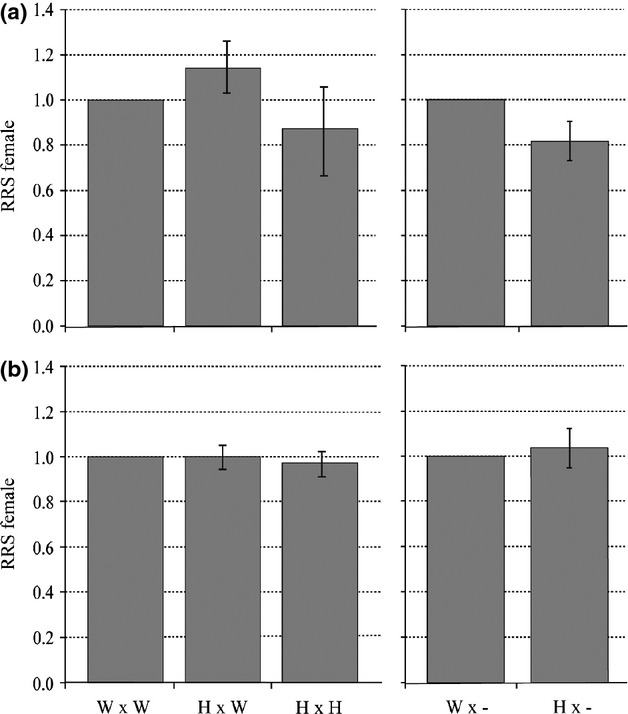
Relative reproductive success (RRS) of each F_1_ mating type in the wild, relative to *W* × *W* or *W* × – (RRS = 1.0, by definition). ‘–’ equals unknown/unassigned parent. (a) Female F_1_s, (b) male F_1_s. Weighted geometric mean RRS among return years 2003–2005 is plotted for *H* × *W* and *H* × *H* relative to *W* × *W* on the left panels, and for *H* × – relative to *W* × – on the right panels. Error bar represents 1 SD.

Comparisons of reproductive success for naturally spawning F_1_ fish by mating type (*H* × *H*, *H* × *W*, *H* × – vs. *W* × *W* and *W* × –) are shown separately for males and females in Table 3 (reproductive success and variance estimates are shown in Table S5, Supporting information). Compared with the fitness of mating by two wild-origin parents (*W* × *W*), the mating by two hatchery-reared parents (*H* × *H*) and one hatchery-reared and one wild-origin (*H* × *W*) parent averaged 94.3% and 107.0%, respectively, for both sexes combined and was not significantly different from 1.0 in any comparison (Table 3; Fig. 4). Although RRS point estimates varied among years for both males and females, they were not significantly different from 1.0 in any comparison (Table 3). Four offspring assigned to *H* × *H* matings in 2003, and RRS of *H* × *H* females relative to *W* × *W* females was 0.87. The small sample size for *H* × *H* matings in 2003 was due to few F_1_ hatchery females returning that year relative to wild, because most of the hatchery females produced in 1998 largely returned as 4-year-olds (65%) in 2002. Table S3 (Supporting information) shows the breakdown of sample sizes by age and sex for fish returning from the two initial supplementation years. Specifically, in return year 2003, there were almost twice as many wild 5-year-old females returning from BY 1998 compared with 5-year-old hatchery females (which largely returned as 4-year-olds in 2002). Removing year 2003 (due to small sample size) in overall estimates of RRS for *H* × *H* vs. *W* × *W* comparisons for males and females revealed similar results to those reported in Table 3 (females: RRS = 0.86, *P* = 0.36, males: RRS = 0.96, *P* = 0.97). Despite small sample sizes for single-parent assignments, comparisons over all years for both sexes (*H* × – vs. *W* × –) yielded similar results where *H* × – produced offspring at 90.5% of *W* × –, which was also not significantly different from 1.0 (Table 3; Fig. 4).

## Discussion

The primary goals of the supplementation programme appear to have been met by providing a demographic boost to the wild population without significantly reducing fitness during the initial two generations of supportive breeding. Hatchery rearing of wild fish resulted in more wild-born adults in the next two generations than if fish had been left to spawn in nature, presumably due to survival advantages conferred by hatchery rearing. We generally fail to reject the null hypothesis that reproductive success of hatchery-reared fish is equal to that of wild-origin fish. The exception of significantly low values of RRS in BYs 2002 and 2003 was driven by hatchery males that did not reproduce, and thus had no effect on fitness of the wild population. Our results show that the reproductive success of successful hatchery-reared parents was not significantly different from wild and that mating types involving hatchery-reared parent(s) (*H* × *H*, *H* × *W*; or *H* × –) were not significantly different from mating by wild-origin parent(s) (*W* × *W*; or *W* × –). Thus, evidence does not support that Chinook salmon reared for a single generation in the hatchery had negative fitness effects on wild-origin fish in Johnson Creek.

Further investigation into significantly low reproductive success of hatchery-reared males compared with wild males in 2 years revealed that this result was largely driven by individuals that produced no offspring: (i) 3-year-old males (jacks) from BY 2000 and (ii) 4-year-old males from the first supplementation year, BY 1998. Low reproductive success of hatchery-reared jacks compared with their wild-origin jack counterparts may be due to differences in rearing conditions, such as increased growth opportunities in the hatchery environment. The incidence of early maturation in hatchery Chinook salmon is higher than in the wild (Larsen *et al*. 2004), as is the case in Johnson Creek. Hatchery-reared jacks from BY 2000 comprised 41% of the F_1_ hatchery returns, whereas wild-origin jacks comprised only 13% of F_1_ wild returns from BY 2000. In general, jacks are at a disadvantage for breeding success compared with large males that have better access to mating with females (Foote *et al*. 1997; Berejikian *et al*. 2010), and the higher incidence of jacks produced in the hatchery may further impact reproductive success compared with their wild-origin jack counterparts. Despite the higher incidence of jacks among hatchery returns, there is no evidence of a shift in age at return for the natural population over time (data not shown). The consequences, if any, of the hatchery jacks on the long-term viability of the natural population will be evaluated in the future.

The lowest values of RRS were observed for age 4 hatchery returns in 2002 (from BY 1998) for both males and females. This result was only statistically significant for males, but RRS estimates were below one for females returning from the first year of supplementation, and power to detect significant differences in these comparisons was low. This result is consistent with Araki *et al*. (2007b), who found that hatchery-reared fish did slightly worse in the first major return year of supplementation. However, the comparisons for females returning in 2004 and 2005 (representing the second year of supplementation, BY 2000) showed RRS estimates >1. High annual variation in RRS of hatchery-origin fish is common in these types of studies (Araki *et al*. 2009), and additional annual comparisons will be needed to better understand the effect of hatchery rearing on the fitness of hatchery females in Johnson Creek.

Many hatchery-reared fish that returned to spawn in 2002 (from BY 1998, age 4) did not produce offspring, and this may be due to density-dependent effects and sexual selection. Return year 2002 had >1000 returning adults, making it the third highest return of Chinook salmon to Johnson Creek, behind only 2001 and 2010. Fleming & Gross (1993) observed hatchery-reared fish to be at a reproductive disadvantage compared with wild fish under high densities, with this effect especially pronounced in males. Density may also have had an effect in 2001 and 2010, but we could only compare the age 3 component (jacks) in 2001 because the eight natural jacks did not produce returning offspring, and in 2010 will not be evaluated until offspring return in 2013 through 2015. Density effects on fitness may result from hatchery-reared males showing less aggression compared with wild males when competing for access to spawning females (Fleming *et al*. 1996; Pearsons *et al*. 2007), possibly an outcome of relaxed selection in the hatchery environment (Theriault *et al*. 2011). Indeed, two studies on the reproductive success of Chinook salmon also showed a stronger effect of hatchery rearing on males than on females (Williamson *et al*. 2010; Anderson *et al*. 2012).

Our study may provide additional support of relaxed selection in the hatchery as a mechanism for reduced reproductive success. Similar to Theriault *et al*. (2011), we found that F_1_ hatchery-reared males had significantly reduced fitness compared with hatchery-reared females, suggesting a role for sexual selection. The reduction in fitness for males may be attributable to the artificial mating of competitively less fit males (e.g. less aggressive) that may not have otherwise successfully reproduced in the wild. In addition, the reduced reproductive success of hatchery males in 2 years may also be influenced by environmental effects in the hatchery.

Reproduction in the natural environment allows an opportunity for selection to act, providing a fitness advantage to individuals that are best suited to the local environment. Although genetic adaptation to captivity can occur rapidly (e.g. Christie *et al*. 2011), it is important to recognize that selection also acts in the natural environment when hatchery-reared fish return to spawn, where only a portion successfully contributes offspring to the next generation. These are the individuals that have the potential to directly impact fitness of the wild population, but we found no evidence of a negative fitness effect on wild fish when hatchery fish mated with them, and this was consistent for both males and females. Reproductive success of *H* × *H* pairings compared with *W* × *W* pairings for 2 of the 3 compared years resulted in RRS <1.0 for females and lower RRS for *H* × – females relative to *W* × – females in all three comparisons. Possible concern is warranted with regard to the RS of *H* × *H* pairings, as they may not produce as many returning adult offspring as *W* × *W* or *W* × *H* pairings.

We found no significant reduction in fitness of the hatchery fish that were successful during reproduction and more importantly, and we found no reduction in the fitness of wild fish when they mated with hatchery fish—a result that is novel compared with other published RRS studies. Araki *et al*. (2007b) found that first-generation hatchery fish (from a traditional hatchery) were reproductively less fit than wild fish and that second-generation wild-born fish produced from two hatchery parents had even lower reproductive fitness, suggesting a carry-over effect of artificial rearing that inflicted negative fitness impacts to wild fish (Araki *et al*. 2009). The lack of prior history of hatchery influence in our system, as evidenced by a lack of hatchery influence detected in Johnson Creek and the Secesh River (unsupplemented) compared with the heavily supplemented upper mainstem of the SFSR (Matala *et al*. 2012), may be an important difference between the hatchery programme evaluated in our study and the systems that have been evaluated in other studies. Domestication impacts from nearby hatchery releases are possible despite the effort to exclude hatchery strays from Johnson Creek; however, those impacts are greatly reduced compared with other systems that are the topic of published RRS studies. Minimal prior hatchery influence in Johnson Creek further increases the potential to detect significant differences in RS between hatchery and wild fish, yet evidence for differences was limited to males that did not produce any offspring. In addition, domestication impacts are further reduced due to the nature of the Johnson Creek supplementation programme as the genetic composition of brood stock represents wild-origin fish that experience their entire life cycle in the natural environment. Minimal domestication impacts in Johnson Creek may help to explain why we did not find that hatchery fish reduced the fitness of wild fish. For example, steelhead in the Hood River system (Araki *et al*. 2007b, 2009) had a history of out-of-basin hatchery influence prior to initiation of their RRS study, and hatchery fish were incorporated into brood stock each year. Similarly, programmes that were the subject of the RRS studies by Williamson *et al*. (2010), Berntson *et al*. (2011) and Theriault *et al*. (2011) also involve hatchery programmes that use brood stock comprised in large part (up to 70–80%) by hatchery-reared fish each year. Indeed, even a few generations of domestication can have negative effects on natural reproduction in the wild (Araki *et al*. 2007a; Christie *et al*. 2011). These empirical studies indicate that use of primarily hatchery-origin fish in brood stock may result in poor performance in the wild (more generations of domestication selection) and may translate to reductions in fitness of wild fish when hatchery-reared fish mate with them.

Our study does not directly estimate genetic versus environmental components of differences between hatchery-reared and wild-origin fish (F_1_s experienced different rearing environments), which would allow us to determine whether there is a carry-over effect of artificial rearing (as found in analysis of F_2_ RRS by Araki *et al*. 2009). However, based on our results thus far, it would be unexpected to see a fitness decline between the F_1_ and F_2_ generations because the F_2_ generation is an additional generation removed from potential domestication effects, and we did not observe fitness declines of wild fish in the F_1_ generation when they mated with hatchery-reared fish. We recognize that even though only wild-origin fish are used as brood stock each year, the effects of hatchery rearing may inflict small changes in fitness that may not result in significant differences in one generation, but the possibility exists for changes to accumulate over time. The effect of supplementation on the natural population over greater than two generations will be evaluated in future years.

Our power to detect significant differences in reproductive success between hatchery-reared and wild-origin fish varied annually and is comparable to published studies where, in some years, a 50% or greater reduction in hatchery-reared reproductive success would be needed to detect a significant difference from wild-origin reproductive success (Araki *et al*. 2007a,b; Theriault *et al*. 2011). Despite some single years with reduced power, combining probabilities across multiple data sets (years) for both single-sex and mating type comparisons did not yield significant results (with the exception of males described above). Further, removal of years with low sample size had no appreciable effect on RRS comparisons. Overall, our study represents one of the most thorough data sets from a wild population to evaluate relative fitness of a supportive breeding programme. This is evident from the number of years (13) included to represent a multiple generation pedigree of spawning adults, number of fish genotyped (7481), number of microsatellite loci (15) and proportion of offspring that were able to be assigned to parents (87%). These numbers compare favourably to other studies of RRS (Araki *et al*. 2007a,b, 2009; Williamson *et al*. 2010; Berntson *et al*. 2011; Theriault *et al*. 2011; Anderson *et al*. 2012).

A variety of management protocols and strategies exist among Pacific salmonid hatchery programmes (Naish *et al*. 2007; Paquet *et al*. 2011), and each species represents multiple genetic lineages and life history traits (Waples *et al*. 2001). Given such diversity, from relatively few and isolated RRS studies conducted so far, it would be premature to generalize that all hatchery-reared fish are significant drivers of fitness declines in wild populations. Specifically, perhaps steelhead, which have been the focus of many RRS studies, are simply more prone to reduced fitness due to hatchery rearing practices. In hatcheries, prior to release in the wild, steelhead juveniles are reared for 1 year until smoltification, a physiological process that prepares fish for transition from freshwater to saltwater. The accelerated smoltification process in the hatchery deviates from the typical 2-year time frame to smolt in nature. Alternatively, Chinook salmon are reared in hatcheries for a time frame more similar to their natal juvenile rearing time of 1 year. Populations experiencing a captive environment that is most similar to what is experienced in the natural environment may show the least divergence from the original wild population (Shuster *et al*. 2005), and risks of genetic adaptation to artificial environments are reduced with fewer numbers of generations in captivity (reviewed in Williams & Hoffman 2009). Nevertheless, our results place into question the generalization that all hatchery fish are significant drivers for fitness declines by demonstrating that supplementation programmes, under certain management practices (e.g. using local wild-origin brood stock, minimal time spent in captivity), can successfully boost population size with minimal negative impacts to the fitness of Chinook salmon in the wild.

In the face of environmental perturbations, fishery harvest and habitat alterations, the ability for anadromous salmonids at risk of extinction to recover to sustainable levels is uncertain. Supportive breeding is simply one of the many tools needed to re-build depressed populations and maintain abundance. In addition to salmonids, many species are incapable of sustaining themselves predominately due to human impacts, and the need to take individuals into a captive environment for long-term survival is a reality for many threatened and endangered species. A goal for captive programmes is to limit deleterious genetic changes during captivity, so that the long-term viability of a population in the wild environment is maximized. One way to minimize the effects of adaptation to captivity, and perhaps subsequent negative impacts on wild populations, is to incorporate some portion of wild genes into the captive population each year. Our study highlights the value in using wild individuals adapted to local environmental conditions for supportive breeding.

## Notes

M.A.H. is a conservation geneticist studying reproductive success of salmonid fishes using parentage based tagging as a tool for conservation and management. C.D.R. is a lead fisheries biologist involved with restoring Chinook salmon. J.L.V. leads research studies contributing to fisheries management. J.J.S. is a conservation geneticist and laboratory manager studying salmon and steelhead. D.D.N. is a fisheries biologist studying salmon. S.R.N. is a conservation geneticist who leads a research group interested in population genetics, local adaptation, and reproductive success of salmonid fishes.

## Data accessibility

Sample locations and microsatellite data: DRYAD entry doi: 10.5061/dryad.19p14.

## Supporting information

**Table S1** Summary statistics for each microsatellite locus. **Table S2** Number of individuals by origin for each return year that were genotyped for this study, and were included in parentage analysis. **Table S3** Relative reproductive success (RRS) of female, male and jack F_1_ fish (including potential parents producing zero adult offspring) from BY 1998 and 2000. **Table S4** Supplementary information for Table 2, showing the proportion of F_1_ fish (from BY 1998 and 2000) that produced one or more returning adult offspring in 2002–2004. **Table S5** Supplementary information for Table 3, showing average reproductive success (RS) and variance estimates. **Fig. S1** Histogram of estimated fitness (i.e. number of offspring produced) for hatchery- and wild-origin female natural spawners from 1998 through 2005.
