# Supplementary material for: Supportive breeding boosts natural population abundance with minimal negative impacts on fitness of a wild population of Chinook salmon
Source: Mol Ecol. 2012 Oct 1;21(21):5236–50. doi: 10.1111/mec.12046 (PMC3490153; doi:10.1111/mec.12046)
Supplement: Supplementary file 1 — Table S1 Summary statistics for each microsatellite locus. Table S2 Number of individuals by origin for each return year that were genotyped for this study, and were included in parentage analysis. Table S3 Relative reproductive success (RRS) of female, male and jack F1 fish (including potential parents producing zero adult offspring) from BY 1998 and 2000. Table S4 Supplementary information for Table 2, showing the proportion of F1 fish (from BY 1998 and 2000) that produced one or more returning adult offspring in 2002–2004. Table S5 Supplementary information for Table 3, showing average reproductive success (RS) and variance estimates. Fig. S1 Histogram of estimated fitness (i.e. number of offspring produced) for hatchery- and wild-origin female natural spawners from 1998 through 2005. [file mec0021-5236-SD1.docx]

Table S1. Summary statistics for each microsatellite locus.  **k is the number of for each microsatellite locus.hat are included in analysisy table to showpe. Therefore, total sample sizes o**k is the number of alleles, HObs and HExp is the observed and expected heterozygosity respectively, NE-P1, NE-P2, and NE-PP represents the non-exclusion probabilities of the first parent, second parent, and parent pair respectively, and F(Null) is the null allele frequency.

Combined non-exclusion probabilities: First parent = 2.30E-07, Second parent = 2.91E-10, and Parent pair = 2.25E-17.

| Locus | k | HObs | HExp | NE-1P | NE-2P | NE-PP | F(Null) |
| --- | --- | --- | --- | --- | --- | --- | --- |
| Ots 212 | 22 | 0.854 | 0.862 | 0.432 | 0.274 | 0.110 | 0.005 |
| Ots 211 | 27 | 0.916 | 0.926 | 0.261 | 0.150 | 0.038 | 0.005 |
| Ogo 4 | 12 | 0.884 | 0.870 | 0.417 | 0.262 | 0.104 | -0.008 |
| Ots 3m | 8 | 0.636 | 0.615 | 0.792 | 0.641 | 0.470 | -0.019 |
| Ssa 408 | 25 | 0.745 | 0.761 | 0.592 | 0.409 | 0.201 | 0.010 |
| Ots 100 | 60 | 0.948 | 0.947 | 0.190 | 0.106 | 0.019 | 0.000 |
| Ots 213 | 31 | 0.929 | 0.928 | 0.254 | 0.146 | 0.036 | -0.001 |
| Ots 474 | 7 | 0.030 | 0.030 | 1.000 | 0.985 | 0.970 | 0.001 |
| Ogo 2 | 12 | 0.785 | 0.777 | 0.597 | 0.418 | 0.227 | -0.004 |
| Ots 9 | 5 | 0.590 | 0.597 | 0.811 | 0.651 | 0.482 | 0.005 |
| Ots 201b | 33 | 0.884 | 0.895 | 0.339 | 0.205 | 0.064 | 0.007 |
| Ots 208 | 31 | 0.935 | 0.939 | 0.220 | 0.124 | 0.026 | 0.002 |
| Omm 1080 | 45 | 0.955 | 0.955 | 0.166 | 0.091 | 0.015 | 0.000 |
| Oki 100 | 24 | 0.927 | 0.933 | 0.238 | 0.135 | 0.031 | 0.003 |
| Ots 311 | 49 | 0.960 | 0.961 | 0.148 | 0.080 | 0.012 | 0.000 |

Table S2. Number of individuals by origin for each return year that were genotyped for this study, and were included in parentage analysis. Genotyping was attempted for an additional 245 fish, but were removed due to an excess of missing data (n=187) or due to duplicate samples (n=58).

| Return year | Hatchery | Natural | Stray | Total |
| --- | --- | --- | --- | --- |
| 1998 | 0 | 131 | 0 | 131 |
| 1999 | 0 | 15 | 0 | 15 |
| 2000 | 0 | 146 | 13 | 159 |
| 2001 | 249 | 1222 | 37 | 1508 |
| 2002 | 428 | 747 | 17 | 1192 |
| 2003 | 189 | 584 | 12 | 785 |
| 2004 | 117 | 204 | 8 | 329 |
| 2005 | 64 | 129 | 2 | 195 |
| 2006 | 72 | 102 | 1 | 175 |
| 2007 | 218 | 161 | 4 | 383 |
| 2008 | 363 | 327 | 22 | 712 |
| 2009 | 518 | 274 | 28 | 820 |
| 2010 | 483 | 556 | 38 | 1077 |
| Total | 2701 | 4598 | 182 | 7481 |

Table S3. Relative reproductive success (RRS) of female, male and jack F_1_ fish (including potential parents producing zero adult offspring) from BY 1998 and 2000.

| Return year | N F1 (H/W) | RS Hatchery | | Variance Hatchery | | | | RS Wild | | Variance Wild | | | | | RRS^+^ | | p-value | | 80%/95% Power ^++^ | | | | | Age of returns | | |
| --- | --- | --- | --- | --- | --- | --- | --- | --- | --- | --- | --- | --- | --- | --- | --- | --- | --- | --- | --- | --- | --- | --- | --- | --- | --- | --- |
| Females |  |  | |  | | | |  | |  | | | | |  | |  | |  | | | | |  | |  |
| 2002 | 167/42 | 0.21 | | 0.26 | | | | 0.38 | | 0.39 | | | | | 0.55 | | 0.08 | | 0.67/0.51 | | | | | 4yr from BY1998 | | |
| 2003 | 87/165 | 0.29 | | 0.32 | | | | 0.34 | | 0.43 | | | | | 0.84 | | 0.60 | | 0.69/0.53 | | | | | 5yr from BY1998 | | |
| 2004 | 39/41 | 2.62 | | 4.51 | | | | 2.05 | | 4.70 | | | | | 1.30 | | 0.26 | | 1.30/1.46 | | | | | 4yr from BY2000 | | |
| 2005 | 8/4 | 4.25 | | 1.07 | | | | 3.75 | | 12.25 | | | | | 1.19 | | 0.77 | | 1.43/1.63 | | | | | 5yr from BY2000 | | |
| Overall female* |  |  | | | |  |  | | | | |  | | 1.00 | | 0.19 | | | | |  | |  | |  |  |
| Males (4- & 5-year-old) |  | |  | |  | | | |  | |  | |  | | | | |  | |  | |  |  |  |  |  |
| 2002 | 255/155 | 0.11 | | 0.15 | | | | 0.26 | | 0.34 | | | | | 0.43 | | <0.01* | | 0.76/0.64 | | | | | 4yr from BY1998 | | |
| 2003 | 29/100 | 0.34 | | 0.59 | | | | 0.38 | | 0.54 | | | | | 0.91 | | 0.89 | | 0.44/0.21 | | | | | 5yr from BY1998 | | |
| 2004 | 42/50 | 1.57 | | 4.20 | | | | 2.28 | | 5.23 | | | | | 0.68 | | 0.13 | | 0.73/0.60 | | | | | 4yr from BY2000 | | |
| 2005 | 0/0 | - | | - | | | | - | | - | | | | | - | | - | | - | | | | | 5yr from BY2000 | | |
| Overall male |  |  | | | |  |  | | | | |  | | 0.64 | | 0.01* | | | | |  | |  | |  |  |
| Jacks (3-year-old) |  |  | | | |  |  | | | | |  | |  | |  | | | | |  | |  | |  |  |
| 2001 | 237/8 | 0.05 | | 0.05 | | | | 0.00 | | 0.00 | | | | | n/a | | n/a | | n/a | | | | | 3yr from BY1998 | | |
| 2003 | 63/16 | 0.29 | | 0.34 | | | | 0.88 | | 1.32 | | | | | 0.32 | | <0.01* | | 0.69/0.51 | | | | | 3 yr from BY2000 | | |
| Overall jack |  |  | | | |  |  | | | | | - | | - | |  | | | | |  | |  | |  |  |

*N* is the sample size for number of naturally spawning hatchery-reared and wild F_1_ fish from BY1998 and BY2000.

*Overall RRS estimate for females does not include return year 2005 due to low sample size.

^+^RRS (relative reproductive success) is calculated as the RS of hatchery-reared fish over the RS of wild-origin fish, and associated *p*-values are based on two-tailed permutation tests. Overall RRS was estimated using weighted geometric means and the according *p*-values were calculated (an asterisk indicates statistical significance).

^++^Statistical power is the RRS value that would be significant with 80% and 95% probability.

n/a is “not available” since wild jacks in 2001 did not produce any adult offspring

Table S4. Supplementary information for Table 2, showing the proportion of F_1_ fish (from BY1998 and BY2000) that produced one or more returning adult offspring in 2002-2004.

| Return year | % successful parent, Wild | % successful parent, Hatchery |
| --- | --- | --- |
| Females |  |  |
| 2002 | 31% | 17% |
| 2003 | 26% | 23% |
| 2004 | 78% | 82% |
| Mean females | 45% | 41% |
|  |  |  |
| Males |  |  |
| 2002 | 21% | 9% |
| 2003 | 28% | 21% |
| 2004 | 72% | 62% |
| Mean males | 40% | 31% |
|  |  |  |
| Jacks |  |  |
| 2001 | 0% | 4% |
| 2003 | 50% | 24% |
| Mean jacks | - | 14% |

Table S5. Supplementary information for Table 3, showing average reproductive success (RS) and variance estimates.

|  | RS hatchery | Variance hatchery | RS wild | Variance wild |
| --- | --- | --- | --- | --- |
| F2 having two hatchery-reared parents: H x H vs W x W | | | | |
| Females |  |  |  |  |
| 2003 | 1.00 | 0.00 | 1.15 | 0.28 |
| 2004 | 1.67 | 1.28 | 2.19 | 2.45 |
| 2005 | 2.50 | 2.27 | 2.20 | 2.18 |
|  |  |  |  |  |
| Males |  |  |  |  |
| 2003 | 1.33 | 0.33 | 1.29 | 0.34 |
| 2004 | 2.00 | 0.84 | 2.14 | 2.40 |
| 2005 | 2.50 | 6.45 | 2.44 | 2.53 |
|  |  |  |  |  |
| F2 having one hatchery-reared parent and one wild-origin parent: H x W vs W x W | | | | |
| Females |  |  |  |  |
| 2003 | 1.21 | 0.23 | 1.15 | 0.28 |
| 2004 | 2.45 | 2.44 | 2.19 | 2.45 |
| 2005 | 2.83 | 2.32 | 2.20 | 2.18 |
|  |  |  |  |  |
| Males |  |  |  |  |
| 2003 | 1.24 | 0.31 | 1.29 | 0.34 |
| 2004 | 2.30 | 2.39 | 2.14 | 2.40 |
| 2005 | 2.27 | 2.62 | 2.44 | 2.53 |
|  |  |  |  |  |
| F2 having one hatchery-reared parent and one missing: H x - vs. W x - | | | | |
| Females |  |  |  |  |
| 2003 | 1.00 | 0.00 | 1.11 | 0.11 |
| 2004 | 1.00 | 0.00 | 1.36 | 0.85 |
| 2005 | 1.20 | 0.20 | 1.40 | 0.30 |
|  |  |  |  |  |
| Males |  |  |  |  |
| 2003 | - | - | - | - |
| 2004 | 1.67 | 1.33 | 1.29 | 0.24 |
| 2005 | 1.00 | 0.00 | 1.33 | 0.27 |


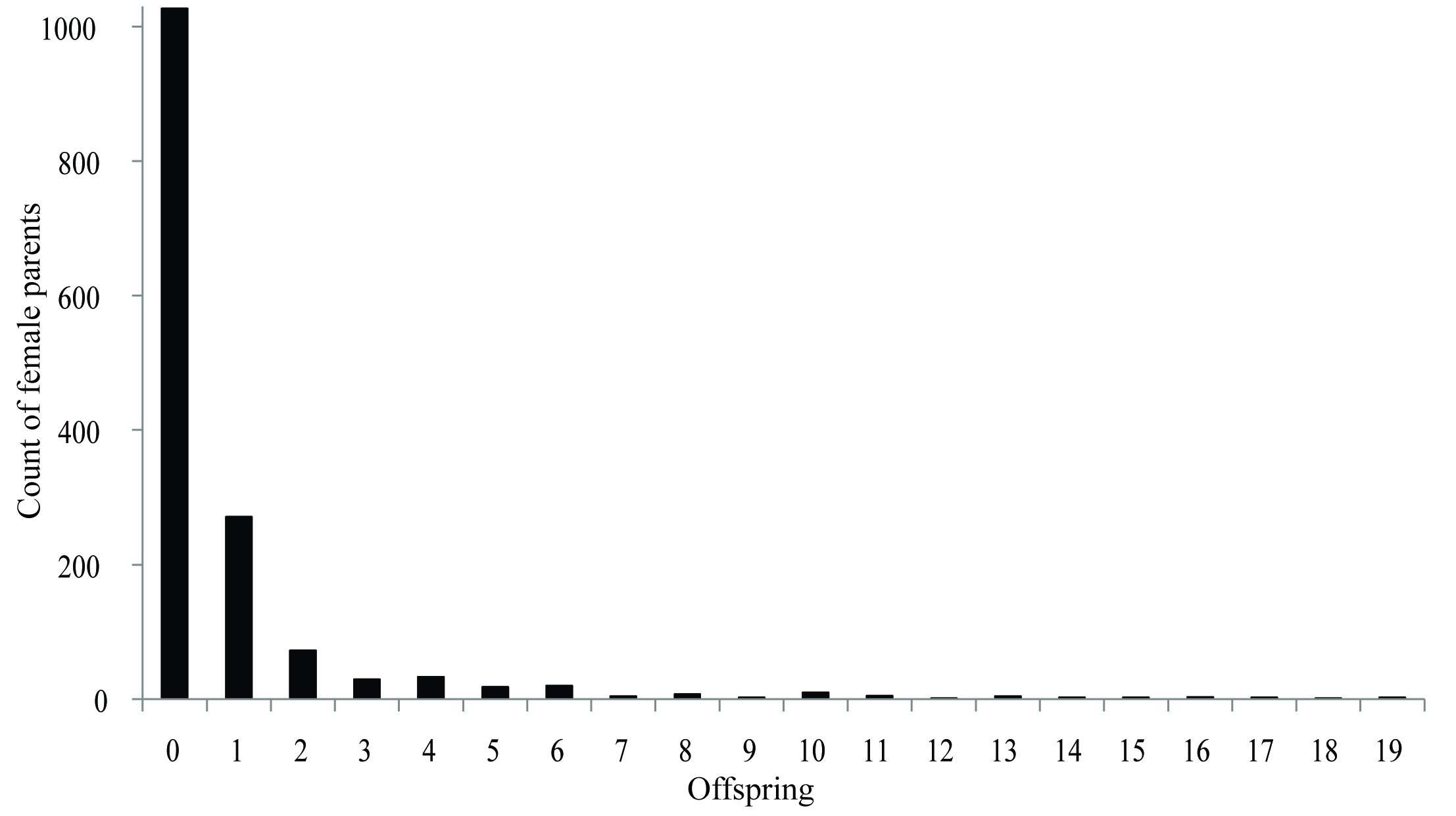


Number of offspring

Figure S1.
